# Measuring Cardiac Interoceptive Accuracy in Infancy: Lessons From the Adult Literature

**DOI:** 10.1111/psyp.70041

**Published:** 2025-03-11

**Authors:** Rosie Donaghy, Matteo Lisi, Jeanne Shinskey, Jennifer Murphy

**Affiliations:** ^1^ Department of Psychology University of Surrey Guildford UK; ^2^ Department of Psychology Royal Holloway University of London Egham UK

**Keywords:** development, infancy, Interoception, interoceptive accuracy

## Abstract

Models of interoception, the processing of internal bodily signals, highlight infancy as a key period for interoceptive learning. Given the potential importance of this developmental period, there has been increasing focus on the measurement of cardiac interoceptive accuracy in infancy. In this paper, we argue that despite progress in this area, the current methods for assessing cardiac interoceptive accuracy in infancy suffer from many of the same limitations as tasks of cardiac interoceptive accuracy employed in adult samples. To progress work in this area, this paper critically reviews methods of cardiac interoceptive accuracy employed in adults and infants and provides several recommendations for optimizing the measurement of cardiac interoceptive accuracy in infants. These include, but are not limited to, methodological choices regarding the presentation of stimuli, the use of control tasks, the analysis strategy employed, and the importance of considering state effects.

## Impact statement

This paper explores the measurement of cardiac interoceptive accuracy in infants, a critical period for interoceptive learning. Despite progress, current infant assessment methods share limitations with adult tasks. To improve research in this field, the paper reviews existing methods and offers recommendations, including refining stimulus presentation, incorporating control tasks, optimizing analysis strategies, and considering state effects to enhance accuracy in measuring infants‘ interoceptive abilities.

## 1 Introduction

Interoception, the processing of the body's internal state, has been associated with a multitude of adaptive psychological processes from emotional ability, feeding and eating behaviors, to social sensitivity and mental and physical well‐being (Brewer et al. 2021). Despite much research into the correlates of individual differences, little research has examined interoception across development (Murphy et al. 2017). Of the handful of developmental studies, changes in interoception have been noted, with improvements in childhood and a decline in late adulthood (Khalsa, Rudrauf, and Tranel 2009; Murphy et al. 2019, 2018). However, one period that remains under‐examined is infancy. This is despite multiple models highlighting infancy as a potentially sensitive period for interoceptive learning, making research into this age group a particular priority (Prentice et al. 2022; Quattrocki and Friston 2014). With growing interest in assessing cardiac interoceptive accuracy in infancy^1^ and the publication of novel methods (Maister et al. 2017), there is a need for scrutiny of the methods used to assess individual differences. Indeed, despite great interest in interoception across the lifespan, the measurement of individual differences—even in adulthood—remains problematic (Brener and Ring 2016; Desmedt et al. 2023; Murphy 2023). With greater focus on optimizing the measurement of interoception, the aim of this paper is to outline important considerations for the measurement of cardiac interoceptive accuracy in adults and illustrate the application of these considerations to interoceptive assessment in infants, to optimize measurement approaches. This paper first outlines the assessment of cardiac interoceptive accuracy in adults and associated challenges, before considering the current tools for assessing cardiac interoceptive accuracy in infants. Drawing on findings from adults, this paper then outlines several practical considerations for improving the measurement of cardiac interoceptive accuracy in infancy.

### 1.1 Measuring Cardiac Interoceptive Accuracy in Adulthood

As reviewed by Desmedt et al. (2023), cardiac interoceptive accuracy in adults is generally assessed using two main task formats—estimation methods or multisensory integration tasks (Brener et al. 1993; Dale and Anderson 1978; Schandry 1981; Whitehead et al. 1977). Multisensory integration tasks most closely correspond to infant measures. In these, participants are required to determine whether an external stimulus (typically a beep or flash) is synchronous with their heartbeat (Whitehead et al. 1977). In the most commonly used 2‐alternative‐forced‐choice (2AFC) format, participants are generally presented with tones at ~200–250 ms (considered to be synchronous) and ~500–550 ms (considered to be asynchronous) after the R wave^2^ and asked to make a binary judgment regarding whether the tones appear to be synchronous with their heartbeat. Whilst scoring methods (e.g., accuracy vs. signal detection measures that separate bias and precision) and instructions (e.g., to determine whether tones are synchronous vs. determine which tones follow heartbeats) vary (Hickman et al. 2020), it has been argued that most 2AFC heartbeat detection variants fail to account for individual differences in preferred delays. Indeed, evidence from multisensory integration tasks with multiple interleaved delays (hereafter “multi‐delay, multisensory integration” tasks; e.g., the method of constant stimuli; Brener and Ring 2016) suggests that there are individual differences in preferred delays. Whilst most individuals perceive signals presented 200–300 ms after the R wave as synchronous with their heartbeat, some individuals (including “interoceptive” individuals—i.e. those with above chance performance^3^) show a preference for other delays (Brener et al. 1993; Clemens 1984; Yates et al. 1985).

It is not known what drives individual differences in preferred delays, though individual differences in the location from which heartbeats are perceived may play a role (see Plans et al. 2021; Brener and Ring 2016). As pulse transit times vary to bodily locations, for example, the chest compared to the finger, if heartbeats are perceived from different bodily locations across individuals, this may contribute to individual differences in the perception of heartbeat‐tone synchrony^4^. While the exact mechanism underpinning individual differences in preferred delays remains unknown, individual differences in preferred delays mean that under certain conditions, the 2AFC task may be liable to false negatives. Simply put, while an individual who scores highly on this task is likely to be interoceptive, individuals may fail this task because the delays used to present stimuli do not align with their preferred delay even though they are truly interoceptive. While this can be somewhat mitigated by examining absolute preferences rather than coding directionality (e.g., by giving participants who display an inverse preference for the “asynchronous” stimuli presented at ~500–550 ms the same score as participants who display the same preference for the “synchronous” stimuli presented at 200–250 ms), any interoceptive individual whose preferred delay falls between these intervals may still respond randomly and be falsely deemed non‐interoceptive. Due to these concerns, newer measures generally present stimuli at multiple delays or use novel analysis strategies (for estimation tasks such as tapping tasks as well as multi‐delay, multisensory integration tasks) and accuracy is inferred from the consistency of a participant's selected delays (Desmedt et al. 2023; Plans et al. 2020; Smith et al. 2021, 2020). Such an approach accounts for individual differences in preferred delays, reducing the likelihood of false negatives.

Whilst these issues have been thoroughly discussed in the literature, several issues remain under examined. This includes the use of control tasks, the influence of state effects, and the external stimulus used in multisensory heartbeat detection tasks (Murphy 2023). With regard to control tasks, evidence from adults has demonstrated that almost 25% of the variance in heartbeat detection ability assessed via multisensory tasks (heartbeat‐tone similarity) is accounted for by general multisensory integration ability (light‐tone similarity; Knapp et al. 1997). Though integration tasks are sometimes used as familiarization procedures or as a screening tool (Brener et al. 1993; Plans et al. 2020), studies of cardiac interoceptive accuracy in adults rarely employ matched control tasks. Whilst a person who scores above chance on any multisensory heartbeat detection task is very likely to be interoceptive, without a matched control task it is not possible to determine whether those who perform poorly on multisensory heartbeat detection tasks do so because they are non‐interoceptive (e.g., they cannot match tones to their heartbeats) or struggle more generally with multisensory integration (e.g., they cannot match two signals from different modalities) or other non‐interoceptive factors (e.g., attention, motivation).

In adults, cardiac interoceptive accuracy is also typically assessed on a single occasion. Whilst this may be sufficient in certain research scenarios, given that only ~40% of the variance in heartbeat detection and estimation tasks can be attributed to trait factors, multiple assessments are required to get an accurate picture of an individual's heartbeat perception (Wittkamp et al. 2018). This may be particularly important where studies seek to manipulate interoceptive ability via training or examine trajectories over time (Murphy 2023). Finally, whilst most adult studies using multisensory heartbeat detection tasks use an auditory external stimulus, some studies employ a visual stimulus. Importantly, only 40% of the variance in visual‐heartbeat multisensory integration appears to be shared with auditory‐heartbeat multisensory integration (Schulz et al. 2013). As such, whether these measures can be used interchangeably remains an outstanding question. Indeed, different tasks of cardiac interoceptive accuracy often do not show strong relationships with each other (Brener et al. 1993; Hickman et al. 2020; Ring and Brener 2018), suggesting that task differences may strongly influence scores—and, as a result, conclusions—obtained.

### 1.2 Measuring Cardiac Interoceptive Accuracy in Infancy

To our knowledge, four studies to date have examined heartbeat perception in infancy using looking preference procedures (Imafuku et al. 2023; Maister et al. 2017; Tünte et al. 2023; Weijs et al. 2023). In the first study by Maister et al. (2017), on each trial infants were presented with a character that pulsed rhythmically either at the same time as the infant's own heartbeat (termed “synchronous” trials) or presented at a rate that was faster or slower than the infant's heartbeat (±10% speed; termed “asynchronous trials^5^”). Each movement was accompanied by a sound to mark the rhythm. For asynchronous trials, an algorithm was used to produce a cardiac‐like rhythm that was ±10% the speed of the infant's average heart rate (HR) recorded from the previous trial (or baseline recording if the first trial was asynchronous). Trials alternated between showing either asynchronous or synchronous movement and appeared on the left or the right of the screen. Infants were shown the character for at least 5 s, after which presentation was contingent on attention. If the infant fixated on the character, it remained for up to 20 s. If the infant fixated away for > 3 consecutive seconds, the trial was terminated. Tasks were terminated when four consecutive trials did not receive sufficient fixation to extend past 5 s, or when the infant became too tired or fussy to continue. A minimum of four synchronous and four asynchronous trials were required for data to be included. Using this procedure (named “iBEATs”) in a final sample of 29 infants aged ~5 months, the authors observed a group preference for stimuli presented asynchronously with heartbeats, though not all infants showed this preference. These results were later replicated by Imafuku et al. (2023) in a sample of similarly aged infants, and a similar study in a sample of Rhesus monkeys also observed a general preference for the asynchronous stimuli (Charbonneau et al. 2022).

Replications were attempted by Tünte et al. (2023) and Weijs et al. (2023). Tünte et al. (2023) utilized this procedure in samples of 3‐, 9‐, and 18‐month‐old infants. In the 9‐month‐old infants, in contrast to the original study, an average preference was observed for synchronous stimuli. The same was observed in the sample of 3‐month‐old infants, though no significant differences were observed at 18 months. Notably, this preference was also not universal, with several infants displaying the opposite preference. In contrast, Weijs et al. (2023) observed no overall preference toward either stimulus in a sample of 31 infants aged 5–7 months. Changes to the testing procedure may account for these discrepant findings; in Tünte et al. (2023) infants always encountered synchronous trials first, whereas the order of presentation was counterbalanced in Maister et al. (2017). Weijs et al. (2023) also made substantial changes to the testing procedure; they included alternating trimodal (audiovisual‐cardiac) and bimodal (visual‐cardiac) blocks, coded behavior manually rather than via eye tracking, and included a grasping task between each trial where infants chose one of two wooden blocks replicating the stimuli seen in the task. However, what is notable is that in all studies individual differences were observed, with some infants displaying a preference for the synchronous stimuli and others showing a preference for the asynchronous stimuli.

### 1.3 Optimizing the Measurement of Cardiac Interoceptive Accuracy in Infancy

Having considered the measurement of cardiac interoceptive accuracy in adults and infants, the following section provides an evaluation of the existing infant tasks and outlines important considerations from the adult literature for optimizing the measurement.

### 1.4 Disentangling Heartbeat Perception From Familiarity Versus Novelty

The iBEATs procedure bears some similarity to the adult 2AFC design in that infants are presented with both synchronous and asynchronous trial types. Importantly, in the original description by Maister et al. (2017), when examining the relationship between task scores and the heartbeat‐evoked potential (a cortical measure thought to reflect the processing of cardiac sensations; see Coll et al. 2021) absolute, not directional, preferences were considered. Simply, infants were deemed to be displaying cardiac interoceptive accuracy if they displayed a preference for either synchronous or asynchronous stimuli. This is a strategy often adopted in infant multisensory integration studies to control for potential individual differences in preferences for familiarity versus novelty or contingency versus non‐contingency (i.e., visual‐tactile contingency detection; Filippetti et al. 2013; Zmyj et al. 2011). Notably, this also aligns with best practice for scoring the adult 2AFC task as described above and should be the scoring strategy used for any study examining the relationship between this task and individual differences variables (see Weijs et al. 2023; Tünte et al. 2023). However, it remains questionable whether this scoring strategy fully accounts for the potential influence of familiarity versus novelty. This is because the iBEATs procedure differs from the 2AFC in some critical ways—there is no consistent asynchronous delay, and asynchronous stimuli are not triggered by the heartbeat. In the original iBEATs task, 50% of trials are synchronous and 50% are asynchronous (±10%). However, asynchronous trials have a more variable speed than synchronous trials (+10% on 25% of trials; −10% on 25% of trials). Indeed, in the original description of the measure, at the group level, synchronous presentations ranged from 376 to 419 ms, whereas asynchronous ranged from 361 to 436 ms (361–392 and 399–436 ms for fast and slow asynchronous trials, respectively). As trials are alternated, it is possible that infants' general preference towards asynchronous stimuli is driven by the greater variation in speed across asynchronous trials. Simply, it may be that infant preferences for synchronous or asynchronous trials do not reflect heartbeat perception, but instead reflect an exteroceptive preference for either familiar stimuli (the more constant speed of the exteroceptive audiovisual stimuli presented in synchronous trial types) or novel stimuli (the more varied speed of the exteroceptive audiovisual stimuli presented in asynchronous trial types). Of course, it is possible that for some infants, HR during the task may be so variable that there is no confound between speed and trial type. For these infants, evidence of an above‐chance preference towards asynchronous or synchronous trial types is likely to reflect perception of heartbeat sensations. However, for infants whose HR is not variable, there may be a confound between trial type and speed (i.e., novelty vs. familiarity).

One method to optimize measurement, ensuring no potential confound between trial type and familiarity versus novelty, would be to utilize a procedure similar to the adult 2AFC task. Instead of creating asynchronous trials from the infant's average HR during the previous trial (as in the iBEATs), like synchronous trials, asynchronous trials could be triggered by heartbeats, but the audiovisual stimuli could be delayed. This method enables audiovisual stimuli to be presented at the same speed across synchronous and asynchronous trials, varying only the synchrony of heartbeat and audiovisual stimuli and avoiding potential influences of familiarity versus novelty. Triggering asynchronous stimuli from the heartbeat also has the added benefit of ensuring that asynchronous presentation does not overlap with actual heartbeats, which may be the case with pre‐recorded stimuli. In terms of scoring, classifying differences in looking times between synchronous and asynchronous conditions as above or at chance, as well as considering the uncertainty surrounding such classifications, as has been recommended in the adult literature (e.g., using Bayesian analyses; Plans et al. 2021), may also help to reduce noise. Indeed, where individual infants do not display a significant difference in looking times to synchronous or asynchronous trials (i.e., performance is at chance), differences in looking times between conditions are not likely meaningful. Using this procedure and scoring strategy, where an infant shows a significant preference towards either synchronous or asynchronous trial types at above chance levels, they are likely to be displaying cardiac interoceptive accuracy with the chance of false positives determined by the statistical threshold.

### 1.5 Consideration of Individual Differences

Whilst the adjusted procedure outlined above is analogous to the adult 2AFC task, as discussed, it has been argued that cardiac interoceptive accuracy in adults is best assessed using multi‐delay rather than 2AFC designs (Brener and Ring 2016). This is due to the observation of individual differences in preferred delays. Whilst a significant preference towards synchronous or asynchronous trials using the adult 2AFC procedure does suggest cardiac interoceptive accuracy, where an individual shows no preference, we cannot conclude that they cannot perceive heartbeats, as it may be that the presentation delays do not align with their preferred delay. Importantly, these concerns are relevant to infancy. Indeed, in all studies using the iBEATS, it is notable that some infants showed a numerical preference for the synchronous stimuli, and others showed a preference for the asynchronous stimuli, which resulted in differential results across studies at the group level (Imafuku et al. 2023; Maister et al. 2017; Tünte et al. 2023; Weijs et al. 2023). Although for the iBEATs procedure, differences in looking times between conditions may be explained by preferences for familiarity versus novelty, as discussed above, it is also possible that individual differences in preferred delays may contribute to variation. Simply, it may be that samples are comprised of infants who vary in their preferred delays either due to chance or due to factors such as age, body morphology (e.g., height, weight) and resting HR, which may influence pulse transit times (also referred to as pulse arrival time) and the locations from which heartbeats are perceived. Crucially, individual differences in preferred delays would also limit the conclusions that could be drawn from the previously described adjusted 2AFC procedure (i.e., the adult 2AFC task adapted for infants); where an infant displays no preference, we would be unable to conclude they are non‐interoceptive, as the delays presented may not align with their preferred delay.

For optimizing the measurement of cardiac interoceptive accuracy in infancy and bringing it in line with best practice, two points are relevant. First, ideally infant multi‐sensory heartbeat detection tasks should present an equal number of trials using at least three delays that are triggered by the R‐wave. This would enable accuracy to be inferred from the consistency of an infant's preferred delays. This procedure, analogous to adult multi‐delay designs, would mitigate the likelihood that an infant performs at chance because the delays used do not align with their preferred delay, thus better enabling us to conclude that those who perform at chance likely do so because they cannot perceive heartbeats. Although some challenges are posed by the inability to instruct infants to focus on one bodily location, as pulse transit times to bodily locations likely vary less in infancy, this is less likely to present a confound and could be accounted for in the following analysis strategy. Second, instead of considering group‐level preferences (that may be influenced by outliers, which is more likely where few trials are available), it may be more informative to consider at the individual level whether infants appear to discriminate between the stimuli (a strategy akin to scoring absolute, not directional, preferences as recommended by Maister et al. 2017). At the most basic level, this could be achieved using an Analysis of Variance (ANOVA) comparing looking times for the three stimulus levels for each infant. Importantly, infants would be considered interoceptive if a significant difference in looking times is observed between any of the three delays, regardless of whether this preference is towards or away. Considering that there may be clusters of preferences (e.g., some infants may look equally at two stimulus delays and less to the third) this analysis would help to account for the possibility that infants may feel their heartbeats from more than one bodily location as well as the possibility that preferred delays may fall between two presented delays^6^.

### 1.6 The Importance of Control Tasks

Whilst the above methods may help us to better conclude that infants who perform at chance are likely not interoceptive, as they mitigate potential effects of familiarity versus novelty as well as delay preferences, there are further non‐interoceptive reasons that an infant may display no preference; they may have difficulties with multisensory integration or other non‐interoceptive factors that may influence looking times (e.g., attention, motivation, muscular control). Whilst few adult studies utilize control measures, as mentioned, almost 25% of the variance in heartbeat‐tone simultaneity judgments is accounted for by light‐tone simultaneity judgments (Knapp et al. 1997). The use of a control task that controls for potential non‐interoceptive effects of multisensory integration processes may be even more important in infant studies as multisensory integration may not be fully developed until 8 years of age (Dionne‐Dostie et al. 2015). Indeed, whilst an infant who shows a significant preference is not likely to have issues with multisensory integration or other non‐interoceptive factors that may influence looking times, without a control task it remains possible that an infant may perform at chance on a heartbeat‐audiovisual task not because they are non‐interoceptive, but because they have general issues with multisensory integration, attention, muscular control, etc., and/or this is not yet fully developed.

Ideally, such a task should be structurally identical to any cardiac interoceptive accuracy task. For example, as the iBEATs task is trimodal (utilizing audio, visual, and heartbeat stimuli), a light vibrotactile stimulus delivered to the infant's foot could be used in place of heartbeats and matched to the infant's average HR recorded from a baseline, ensuring that HR and heart‐rate variability are controlled for. Importantly, where consistency analyses demonstrate that an infant shows no preference for the delays used to present stimuli in the control task, scores from the heartbeat variant are uninterpretable, as an infant may have cardiac interoceptive accuracy but perform at chance due to general difficulties with multisensory integration or other non‐interoceptive factors that may influence looking times.

## 2 Further Considerations

### 2.1 Analysis Strategy

Above we have suggested that at the most basic level individual looking preferences using multi‐delay variants may be examined using a simple ANOVA; however, a more sophisticated analysis strategy may be beneficial (as argued for infant studies more broadly; Byers‐Heinlein et al. 2022). While many techniques are likely to be useful, here we focus on two examples to illustrate benefits. First, as the number of trials may vary between infants, which may contribute to greater sampling variability and extreme data points at the individual level, multi‐level models may be beneficial (Pinheiro and Bates 2000). By accounting for variability at multiple levels (e.g., within and between participants) these models naturally account for these issues, such that scores from participants with fewer trials carry less weight in the estimation of group‐level statistics. Such an approach also allows all data to be retained in models, meaning that an arbitrary cut‐off (e.g., 4 trials per condition) is not required, which would also likely improve statistical power, a frequently cited issue in infant research (Savalei 2021).

Second, more sophisticated modeling may also be useful for addressing other challenges. Whilst discussed in detail by Savalei (2021) and Lindsay and Mather (2022) one example is the importance of modeling within‐participant variability. For example, analyses of tasks of cardiac interoceptive accuracy—and visual preference tasks used in infancy more broadly—generally assume that preferences (e.g., to familiarity vs. novelty) remain stable throughout the task. However, evidence suggests that preferences can change during the course of experimental paradigms (Houston‐Price and Nakai 2004; Roder et al. 2000). To our knowledge, the stability of preferences for synchronous or asynchronous stimuli has not been tested for cardiac interoceptive accuracy as assessed in infancy. It is therefore plausible that an infant may initially show a preference for synchronous stimuli that switches to a preference for asynchronous stimuli or vice versa. This may be even more likely if a state change in HR occurs, as state changes in HR have been correlated with changes in pulse transit time in early infancy (Galland et al. 2007). An infant whose preference changes during the task would likely appear non‐interoceptive, but their looking behavior is not random. This potential within‐participant variability could be modeled using methods that can detect latent change points such as hidden Markov models (Visser and Speekenbrink 2022). This would allow us to test whether our assumptions regarding random responding are correct. Indeed, if we assume that looking preferences remain stable throughout the task and within‐subject variability is observed, it remains a possibility that some infants will be falsely deemed non‐interoceptive when their looking behavior is not random. Although the limited number of trials per infant may make this analytical approach challenging in practice, and a proof of concept that this is feasible is required, these examples serve to illustrate the broader conceptual point that the current analysis methods may not be sufficient for understanding the complexity of these data and that other methods—discussed here and by Savalei (2021) and Lindsay and Mather (2022)—may be more informative if shown to be feasible.

### 2.2 Adapting Measures for Use in Infancy

Whilst employing multi‐delay tasks mitigates the issue of individual differences in preferred delays, it should be noted that the delays used for infant studies should not be the same as those used in adults, as infants have a faster resting HR than adults. This creates a shorter inter‐beat interval (IBI) and risks that stimuli overlap with two heartbeat cycles (i.e., that an asynchronous delay for the preceding heartbeat is a synchronous delay for the following heartbeat). As infants cannot be instructed to attend to stimuli that follow heartbeats, ensuring that selected delays are closer to one heartbeat is essential. Equally, as IBIs vary due to heart‐rate variability, care must be taken to ensure that delays also account for such variability. Whilst the exact delays selected may depend on infant age (as resting HR varies from ~100 to 150 bpm in the first year of life; Ostchega et al. 2011), consider the following worked example: if the infant HR is a maximum of 120 bpm, this creates an IBI of 500 ms. Delays of 0, 100, and 200 ms ensure that the longest delay (200 ms) is still closer to the preceding heartbeat than the following one (i.e., 300 ms after the longest delay). Although it is not known which delays infants can discriminate, evidence from adults suggests the specificity of discrimination (standard deviation of the interval perceived to be synchronous) ranges from 43 to 167 ms (Brener and Kluvitse 1988), making 100 ms bins (the same as those used for the Method of Constant Stimuli; see Brener and Ring 2016) a reasonable starting point. Whilst standardizing this procedure will require further research, this adaptation is likely to be more informative than using variable speed for asynchronous stimuli as in the iBEATs.

### 2.3 State Effects

Whilst state effects are not routinely considered for adults, they may be particularly important for longitudinal developmental studies. Indeed, if only 40% of the variance in any given testing session in adults can be attributed to trait factors, this means that test–retest reliability (not yet reported for infant interoceptive accuracy tasks, though recommended; Savalei 2021) may be particularly low, making single assessments unsuitable for developmental studies examining potential change over time (Savalei 2021; Wittkamp et al. 2018). The use of multiple measures assessing similar processes, recommended for infant research more broadly (Havron 2022), may also help to get a fuller picture of an infant's interoceptive ability (e.g., see Tünte et al. 2023). Whilst multiple assessments pose challenges for infant research, such an approach is crucial for enabling strong conclusions to be drawn. Future research exploiting digital technology suitable for infants, as has been employed in adults (Plans et al. 2020), may help to overcome potential challenges in the future, enabling easier collection of multiple assessments. Where it is not possible to complete multiple assessments at the same developmental stage, studies should endeavor to record and examine the contribution of state‐level factors that show developmental change (e.g., resting HR; Fleming et al. 2011) given that many of these factors have been associated with individual differences in cardiac interoception in adults (see Murphy et al. 2017; Brewer et al. 2021).

### 2.4 Are We Measuring the Same Processes in Infancy and Adulthood?

Finally, it is worth considering that it is not known whether infant measures of cardiac interoceptive accuracy—or childhood more broadly (e.g., Schaan et al. 2019)—measure similar processes to tasks used in adults. Indeed, research in adults has shown that tasks that purport to measure similar processes often show only small‐to‐moderate relationships with each other, rarely meeting the criteria for convergent validity (Brener et al. 1993; Hickman et al. 2020). Importantly, newly developed infant tasks differ from adult tasks in multiple ways. As argued by Murphy et al. (2017), to fully support longitudinal developmental research in this area, it is essential for future research in adults to examine the extent to which implicit measures (e.g., looking preferences) tap similar processes to explicit judgments. Such work should also establish whether the presence of multiple exteroceptive stimuli (e.g., both audio and visual as opposed to a single exteroceptive stimulus typically used in adults) influences scores. It is conceivable that the use of multiple exteroceptive stimuli may influence scores. Indeed, evidence from adults discussed above suggests only 40% of the variance in visual‐heartbeat multisensory integration is shared with auditory‐heartbeat multisensory integration (Schulz 2013). Likewise, studies of multi‐sensory integration in adults suggest different discrimination thresholds (e.g., just noticeable differences) for the perception of vibration‐tone synchrony and light‐tone synchrony, with a larger range of delays for light‐tone synchrony (Knapp et al. 1997). Beyond influencing the convergence between adult and infant tasks, this may also influence the appropriateness of the delays used for infant studies where the perception of audiovisual‐cardiac synchrony is examined. Exploring the former could be achieved by adapting implicit infant tasks for use in adults using similar eye‐tracking procedures (e.g., see Imafuku et al. 2023) and examining the relationship between these measures and explicit measures of cardiac interoceptive accuracy (e.g., multi‐delay tasks) in an adult sample. Although this approach may need to account for potential differences in gaze behaviors between adults and infants (such as the influence of bottom up vs. top down factors; Franchak et al. 2016), such work is likely to be informative. Specifically, this may help to elucidate whether there is a functional significance to infant preferences towards synchronous versus asynchronous stimuli, something that may not be fully accounted for by individual differences in preferred delays, as well as determine whether measures of cardiac interoceptive accuracy in infants measure “accuracy” at all.

### 2.5 Feasibility

Although the above recommendations are aimed at strengthening the inferences that can be drawn from studies of cardiac interoceptive accuracy in infancy, it should be acknowledged that implementing some of these recommendations may be challenging. While triggering the presentation of audiovisual stimuli in asynchronous trials from the infant's heartbeat (plus a delay) would not increase overall testing time, including an additional delay and a control task would increase overall testing time. Examination of state effects would also require multiple visits. Although previous work has demonstrated feasibility after increases in task length (e.g., increasing the maximum number of attempted trials from 48 to 80; Tünte et al. 2023; Maister et al. 2017) and the inclusion of additional tasks (e.g., the iBREATH procedure to assess respiratory interoceptive accuracy alongside cardiac interoceptive accuracy; Tünte et al. 2023), suggesting that the aforementioned recommendations are feasible, an increase in the overall length of testing sessions would make examination of other facets using lengthy behavioral tasks (e.g., emotion, temperament) alongside cardiac interoceptive accuracy challenging.

There are several ways to mitigate these challenges to enhance feasibility. First, in the iBEATs procedure, stimuli are presented for a maximum of 20 s if fixation occurs for more than 5 s. However, average looking times are generally shorter (on average ~6–10 s; Tünte et al. 2023; Weijs et al. 2023). Considering this, one potential option is to terminate fixation after 10–15 s to reduce overall task length. Second, multiple trials are typically used for the iBEATs task (e.g., 48–80 trials; Tünte et al. 2023; Maister et al. 2017). Setting a minimum number of trials and terminating the task when this threshold is reached may also reduce overall task length for some infants. While this would mean variation in task completion time across infants, these differences are inherent to the infant‐controlled procedure (which minimizes fatigue and allows adaption for different age groups) and could be controlled for statistically so as not to introduce an additional confound. Finally, it is possible that after methodological studies are conducted in infants to assess the contribution of potential confounds and individual differences (preferred delays, state effects, multisensory integration) to cardiac interoceptive accuracy scores, it may be found that concerns raised from studies of cardiac interoceptive accuracy in adulthood do not apply to studies of cardiac interoceptive accuracy in infancy; for example, if there is little evidence of state effects in infancy, testing cardiac interoceptive accuracy on one occasion may be shown to be appropriate. Likewise, if few infants perform at chance on the multisensory integration control task, it may be unnecessary to include this in future studies. Similarly, if no infants are found to have a preference for the intermediate stimuli, a 2AFC design may be appropriate. However, without this methodological work, the contribution of these potential confounds to the assessment of cardiac interoceptive accuracy in infancy remains unknown. It is therefore essential that such work is completed, and resulting recommendations implemented, to strengthen the inferences that can be drawn.

## 3 Conclusion

In conclusion, whilst many recent papers have focused on methods for improving infant research more broadly (Havron 2022; Lindsay and Mather 2022; Savalei 2021), arguing for greater consideration of reliability and increased trial numbers, amongst other suggestions, this paper outlined important considerations for the measurement of cardiac interoceptive accuracy in infancy specifically. It was argued that existing approaches have limitations and research in this area would benefit from adapting measures used in adults that account for individual differences in preferred delays and associated analytic approaches. The need for control tasks and the influence of state effects, relevant also for adult studies, were also outlined as important considerations for infant research, with these considerations potentially more important for research on infants compared to adults. Finally, it was argued that to support developmental research in this area, establishing convergent validity between infant and adult tasks will enable stronger conclusions to be drawn. Overall, whilst infant interoception research should adopt existing general suggestions for improving infant research, exploiting the existing knowledge from the adult literature is likely to improve assessment in this area, leading to more reliable infant interoception research.

## Author Contributions

**Rosie Donaghy:** writing – review and editing. **Matteo Lisi:** writing – review and editing. **Jeanne Shinskey:** writing – review and editing. **Jennifer Murphy:** conceptualization, supervision, writing – original draft, writing – review and editing.

## Disclosure

J.M. has completed paid consultancy work for Healios for work on interoception in adults.

## Conflicts of Interest

The authors declare no conflicts of interest.

## Data Availability

The authors have nothing to report.

## Endnotes

^1^
Note that studies often refer to “interoceptive sensitivity” in infancy, rather than accuracy. In the adult literature, interoceptive accuracy is typically defined as the ability to correctly perceive internal signals as measured by objective tests, such as those described in this paper. We use the term accuracy here for consistency with the broader adult literature but note that these two terms are often used interchangeably to refer to measures that provide some evidence of the ability to perceive signals. What terminology is most appropriate for studies of interoception in infancy warrants further consideration but goes beyond the scope of the current paper.

^2^
The largest deflection on an electrocardiogram that represents depolarization of the ventricles, which corresponds to the start of the contraction of the ventricles.

^3^
We use the terms “interoceptive” and “non‐interoceptive” for consistency with the broader literature. When we say that an individual is “interoceptive” we are referring to performance that is above chance on a particular task, at a particular point in time. Where performance is at chance, an individual would be deemed non‐interoceptive on this particular task, at this particular point in time. This terminology is similar to terminology typically used in the field (e.g., “heartbeat perceiver”; Brener and Ring 2016).

^4^
Whether perception from certain bodily locations is strictly internal (visceral) or occurs via external (somatosensory) signal processing (e.g., the vibration of the heart against the chest wall; see Murphy et al. 2017) is difficult to determine without the use of experimental intervention (e.g., topical anaesthetic on the chest; see Khalsa, Rudrauf, Feinstein, et al. 2009). However, most contemporary models of interoception do not restrict to specific physiological pathways for perception to be considered interoceptive, acknowledging that the processing of internal states is underpinned by multiple sensory systems (Desmedt et al. 2023).

^5^
Note that terminology differs across the adult and infant literature. In the adult literature, asynchronous trials typically refer to stimuli that are presented at the same frequency as the current heartbeat, but out of phase. In contrast, in the infant literature this generally refers to stimuli that do not have contingency with the current heartbeat as tones are presented at rate that is faster or slower than a recording of the heartbeat from the previous trial.

^6^
Notably, whilst this method mitigates the possibility that an individual's preferred delay falls between two presented delays, how much stimuli need to be delayed from an individual's preferred delay for stimuli to appear asynchronous (i.e., the just noticeable difference) remains to be established for both adults and infants (though see Brener and Kluvitse 1988). As such, any method that selects multiple delays for presentation will mitigate this possibility (e.g., the use of a 6AFC task rather than a 2AFC task; see Brener and Ring 2016) but may not eliminate it. For a task in adults that does eliminate this possibility, but cannot readily be adapted for infants, see Plans et al. (2021).
